# Bonding of Gold Nanoclusters on Graphene with and without Point Defects

**DOI:** 10.3390/nano10112109

**Published:** 2020-10-23

**Authors:** Theodoros Pavloudis, Joseph Kioseoglou, Richard E. Palmer

**Affiliations:** 1 College of Engineering, Swansea University, Bay Campus, Fabian Way, Swansea, SA1 8EN, UK; tpavlo@auth.gr; 2 School of Physics, Faculty of Sciences, Aristotle University of Thessaloniki, GR-54124 Thessaloniki, Greece; sifisl@auth.gr

**Keywords:** graphene, gold, Au, nanoparticles, nanoclusters, defects, vacancies, ab initio, DFT

## Abstract

Hybrid nanostructures of size-selected nanoparticles (NPs) and 2D materials exhibit striking physical and chemical properties and are attractive for many technology applications. A major issue for the performance of these applications is device stability. In this work, we investigate the bonding of cuboctahedral, decahedral and icosahedral Au NPs comprising 561 atoms on graphene sheets via 10^3^-atom scale ab initio spin-polarized calculations. Two distinct cases we considered: (i) the Au NPs sit with their (111) facets on graphene and (ii) the NPs are oriented with a vertex on graphene. In both cases, we compare the binding energies with and without a graphene vacancy under the NP. We find that in all cases, the presence of the graphene vacancy enhances the bonding of the NPs. Significantly, in the vertex-on-graphene case, the binding energy is considerably increased by several eVs and becomes similar to the (111) facet-on-graphene case. The strain in the NPs is found to be minimal and the displacement of the carbon atoms in the immediate neighborhood of the vacancy is on the 0.1 Å scale. The work suggests the creation of stable NP-graphene systems for a variety of electronic, chemical and photonic applications.

## 1. Introduction

The unique physical and chemical properties of 2D materials—graphene being the primary example—and of nanoparticles (NPs) can be further enhanced by combining the two in hybrid nanostructures. Indeed, NP-graphene composites show promise for electrochemical energy applications such as lithium batteries, supercapacitors and catalysts for various reactions including oxygen reduction, oxygen evolution and hydrogen evolution reactions [1] and a variety of bioapplications, including electronic, electrochemical and optical biosensors, bioimaging, photothermal therapies, drug delivery and tissue engineering [2]. Amongst NPs, Au is attractive for both bio [3,4] and chemical applications [5,6] due to its stability, biocompatibility and catalytic activity, and has been a well-established model material in nanocluster science for decades. During the past decade, several Au NP-graphene hybrid nanostructures have been developed and used in optoelectronic and sensing applications [7,8,9,10].

The triangular lattice of the (111) surface of the face-centered cubic (fcc) metals is a good match for the hexagonal honeycomb lattice of graphene, and the metals are accommodated on graphene with minimal strain depending on their lattice constant. An ab initio investigation reported weak bonding between graphene and Al, Ag, Cu, Au and Pt, with a binding energy of 0.03 eV per carbon atom for Au [11], and large equilibrium separations. Further experimental studies of Au-plated graphene, combining optical microscopy and electronic measurements, showed that the presence of Au on graphene does not affect its electronic structure, electron–phonon coupling and doping levels [12]. A deeper investigation of the dynamics of Au NPs on carbon nanostructures, specifically multiwalled carbon nanotubes (MWNT), showed that they are governed by the van der Waals and electrostatic interactions, with the former being responsible for stabilizing the NPs [13].

The bonding of noble-metal nanoclusters on carbon surfaces has been shown to be enhanced by surface modifications. In Ref. [14], Ag_2700_ clusters produced via a magnetron sputtering, gas aggregation cluster beam source were deposited on highly oriented pyrolytic graphite (HOPG) predecorated with surface defects created by Ar^+^ sputtering. The subsequent scanning tunneling microscopy (STM) investigation revealed a stable, randomly dispersed array of the clusters. The effect of surface modifications on the anchoring of Au NPs on carbon surfaces was showcased in Ref. [15] where small tunnels on the graphite support, created via the implantation of small nanoclusters, immobilized larger Au clusters with sizes from 147 to 923 atoms. This concept was further explored in Ref. [16] using molecular dynamics simulations. The icosahedral Au NPs were immobilized on the graphite nanotunnels. Their shape did not change and the (111) facets of the NPs, oriented parallel to the graphite substrate, did not undergo structural reconstructions, apart from a small region close to the tunnel. Finally, in Ref. [17] researchers investigated the stabilization of very small Au clusters (1 to 39 atoms) on graphene via Density Functional Theory (DFT) calculations and reported a strong interaction and improved bonding when the clusters were sitting on a graphene vacancy. 

In this work, we aim to overcome previous theoretical studies in terms of completeness and size. We employ ab initio calculations of a scale not previously reported in the literature with realistic models to examine and quantify from first principles, the effect of surface modifications, specifically vacancy defects, on the bonding, energetics and structure of Au NP-graphene hybrid nanostructures. We attempt a thorough examination of the system and check for three highly ordered NP structural motifs and two different cluster orientations. We take into account both spin-polarization and van der Waals interactions. Our results show that the creation of vacancies strengthens the bonding of the Au NPs on graphene, particularly when the NPs sits with a vertex on the support. The resulting nanostructures show a symmetrical displacement of the C atoms surrounding the vacancy and minimal strain in both the NPs and the graphene sheet. The results of this work can be expanded to other NP sizes and cluster materials and exploited for the creation of stable and efficient NP-graphene devices.

## 2. Materials and Methods 

The spin-polarized DFT calculations were performed using the Vienna Ab initio Simulation Package (VASP 5.4.4, Vienna, Austria) [18,19] under the Perdew-Burke-Ernzerhof derivation of the Generalized Gradient Approximation (GGA-PBE) [20], with Projector Augmented-Wave (PAW) pseudopotentials [21,22]. The energy cut-off of the plane-wave basis set was 400 eV. The Brillouin zone was sampled at the Γ point and the break condition for the self-consistent electronic loop was 1 meV. The width of the vacuum surrounding the nanoclusters was equal to 15 Å in all directions, which ensured that there was no interaction between the nanoclusters and their nearest image or the graphene sheet above them. We instructed VASP to print the atomic charge densities and magnetization densities in the output file using the default Wigner-Seitz radii for each atom type. Since there is no unambiguous way to determine charge densities, the charge-related results are qualitative in nature, but still valuable in the extraction of charge transfer trends in our examples.

**Figure 1:**
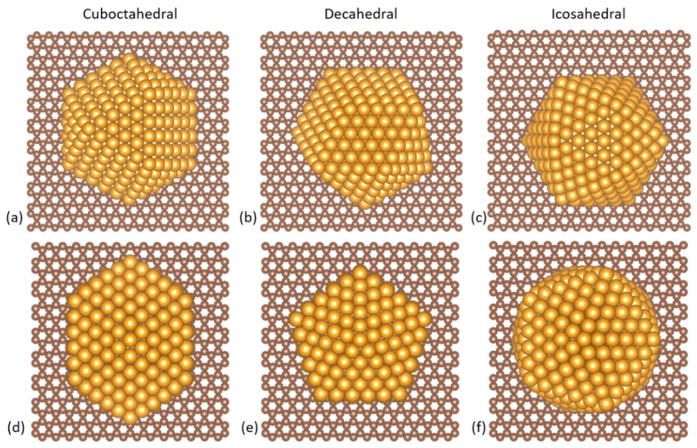
Top-down projections of the Au_561_ NPs on graphene. First row: the (**a**) cuboctahedral, (**b**) decahedral and (**c**) icosahedral NPs with a (111) facet on graphene. Second row: the (**d**) cuboctahedral, (**e**) decahedral and (**f**) icosahedral NPs with a vertex on graphene.

We focus on 561-atom nanoclusters that lie in the catalytically active regime. Such clusters are routinely produced by cluster deposition methods such as the gas-phase condensation method. Choosing a “magic number” for the nanoclusters also allows for a ready comparison between the three highly ordered structural motifs, the cuboctahedral, the Ino-decahedral, and the icosahedral, which are shown to be dominant for Au NPs of this size [23]. We place these NPs on a 576-atom graphene sheet for two distinct cases: (i) where the Au NPs sit with their (111) facets on graphene and (ii) where the NPs are oriented with their vertex on graphene (Figure 1). In both cases, we compare the bonding with and without a graphene vacancy under the NP. We also check for the energetically preferable position of the vacancy under the (111) cluster facet.

Van der Waals interactions were taken into account through the DFT-D3 method [24] with Becke-Jonson damping [25]. Under these settings, the lattice constant of the Au unit cell was found to be 4.101 Å and the *a* lattice constant of the graphene unit cell was found to be 2.468 Å. The choice of the correction method was made based on the results of Ref. [26] on the comparison of different van der Waals corrections to the bulk properties of graphite. The inclusion of van der Waals interactions is an important part of this work. They are known to play a role in the adsorption of small aromatic molecules on Au surfaces [27], the catalytic selectivity for the oxidation of organic molecules on metallic Au [28], the electronic and energetic properties of small Au NPs [29], and to be competing with covalent and ionic bonds for bonding on Au–S surfaces and Au NPs [30]. 

The imaging of the results was performed with the VESTA 3.4 visualization program (Tsukuba, Ibaraki, Japan) [31]. 

The dataset generated during the current study is available online at the Zenodo repository [32].

## 3. Results

### 3.1. Nanoclusters with a Facet on Graphene

**Figure 2:**
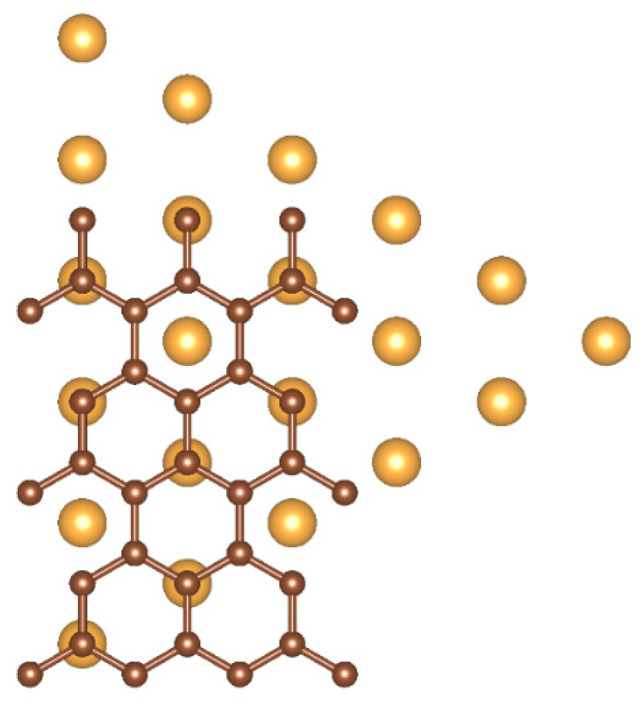
Top-down view of the ideal interface between the (111) triangular facet of the Au_561_ NPs and graphene.

The (111) facets of the NPs were interfaced with graphene following the energetically favorable configuration of according to Ref. [12]: the atoms of these 21-atom triangular (111) facets are accommodated alternately on top of C atoms and in the middle of C hexagons. This particular configuration forces a lattice constant of 4.031 Å on the gold lattice-down from 4.101, Å for an initial strain of only 1.71% for a perfect pairing of the two surfaces (Figure 2).

**Figure 3:**
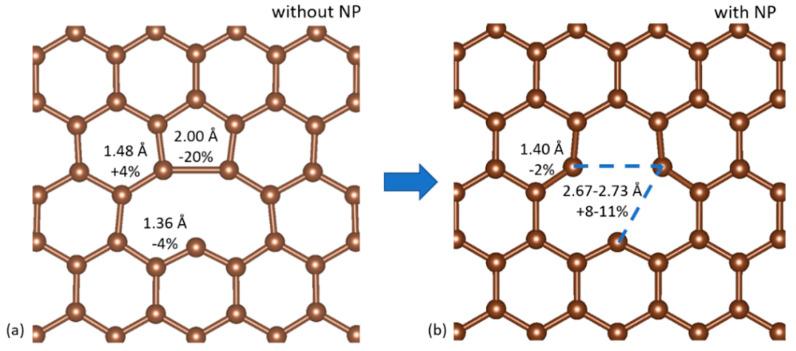
(**a**) The Jahn–Teller distortion of graphene vacancies and (**b**) its removal caused by the presence of the NP vertex. The bond lengths and their relative change compared to pristine graphene are noted.

The most common and energetically favorable point defect of graphene is the single vacancy. We calculated the formation energy $E_{f,vac}$ for the neutral-charge vacancy according to:

(1) $$E_{f,vac}=E_{tot,vac}-E_{tot,sheet}$$

where $E_{tot,sheet}$ is the total energy of the 576-atom pristine graphene sheet and $E_{tot,vac}$ is the total energy of the graphene sheet with the vacancy. We found a formation energy of 7.71 eV, in good agreement with previous studies [33]. The C atoms surrounding the vacancy showed the expected Jahn-Teller distortion, displayed in Figure 3a: two of the three atoms previously bonded to the missing C atom form a bond with a length of 2.002 Å, reduced from their initial distance of 2.468 Å in pristine graphene. The third atom moved further away. We found no out-of-plane displacements of the three3 C atoms. The two C atoms in the pair show magnetic moments of 0.06 $\mu_{Β}$, the third atom shows a magnetic moment of 0.50 $\mu_{Β}$. The charge is unevenly distributed between the three C atoms, with the two in the pair showing a charge of 2.40 e and the third a charge of 2.58 e.

**Figure 4:**
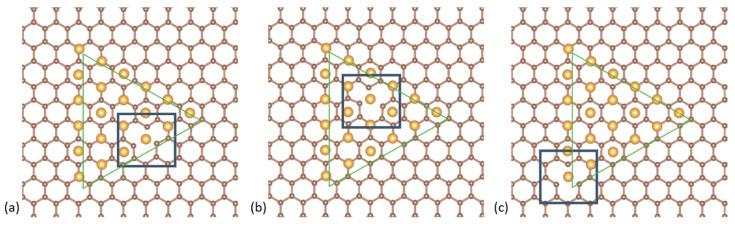
Cross-sections of the top-down projection for the three different positionings of the vacancy under the triangular (111) facet of the NPs: (**a**) under one edge of the triangle, (**b**) near the center of the triangle and (**c**) under a vertex of the triangle (relaxed models).

After the initial set of relaxations of the three isomers on pristine graphene, we investigated the energetically preferred position of the vacancy under the (111) facet of the Au_561_ clusters. To this end, we ran a set of relaxations for three distinct locations of the vacancy under the triangular (111) facet of a cuboctahedral NP: (i) vacancy under one edge of the triangle, (ii) vacancy under a vertex of the triangle and (iii) vacancy near the center of the triangle. These positions are shown in Figure 4. We found that the lowest energy position is below the center of the (111) triangle, followed by the triangle edge, with an energy difference between the two of only 0.01 eV. The vacancy under the cluster’s (111) facet vertex is 0.43 eV higher in energy. Based on these results, we proceeded with the relaxations of the different NP isomers with a vacancy positioned under the center of their (111) facet.

The binding energies $E_{binding}$ were calculated according to:

(2) $$E_{binding}=E_{graphene+particle}-E_{particle}-E_{graphene},$$

where $E_{particle}$, $E_{graphene}$ and $E_{graphene+particle}$ are the total energies after the relaxations for the free NP, the graphene sheet (with or without the vacancy), and the NP-graphene combination, respectively. The results are shown in Table 1. The binding energy followed the icosahedral > decahedral > cuboctahedral order for both substrate configurations. We observed a general increase of the binding energy when the substrate vacancy is added on the scale of 0.17–0.24 eV for all the NPs. 

In pristine graphene, all C atoms present the same charge with only minimal changes in the scale of 0.01 e for the atoms in the proximity of the NP facet. No magnetism appears in any of the atoms of the model. In the defected graphene case, the magnetic moments of the three C atoms surrounding the vacancy do not change considerably. They are reduced by 0.01 $\mu_{Β}$ for the lone C atom and by 0.03 $\mu_{Β}$ for the C atoms in the pair. The charge distribution among these atoms is also greatly unaffected by the presence of the NP: the charge is only 0.01 e lower for the pair and the same as before for the lone C atom.

### 3.2. Nanoclusters with a Vertex on Graphene

**Figure 5:**
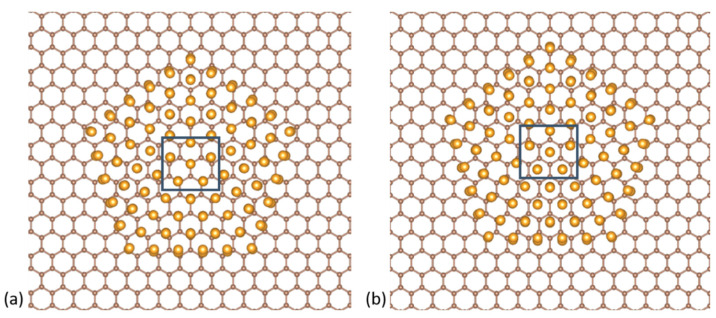
Top-down views of the two different positionings for a decahedral NP on graphene with (**a**) the vertex on top of a C atom and (**b**) on the center of the hexagon formed by the C atoms.

In the case where the cluster vertex points at the graphene substrate, we initially investigated the bonding site of the NPs with a vertex on pristine graphene and ran relaxations for two distinct positions: (i) the vertex of the NP sits on top of a C atom and (ii) the vertex of the NP lies in the center of the C-atom hexagon (Figure 5). We found that the first case is energetically preferable by 0.33 eV, and proceeded with this particular positioning.

**Figure 6:**
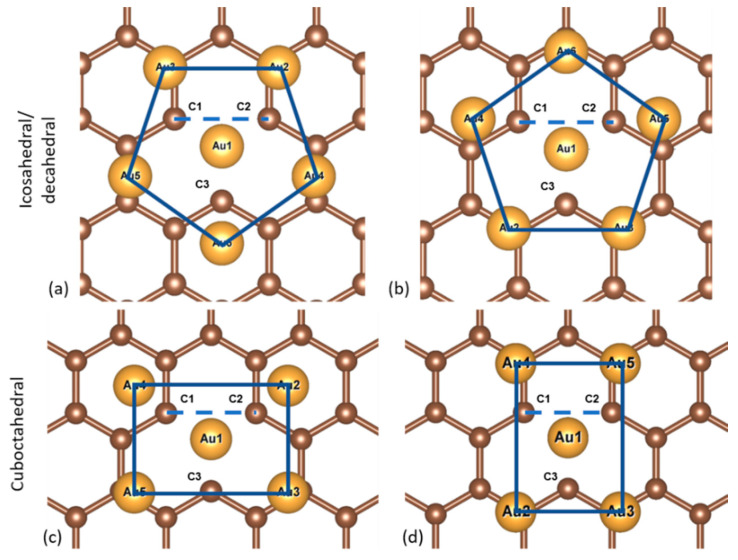
Cross-sections of the top-down views showing the first and second atom layers of the Au_561_ NPs of of the three structural motifs in their different orientations on the graphene vacancy. First row: the vertices of the icosahedral and decahedral NPs with the tip of the pentagon pointing (**a**) towards the lone C3 atom and (**b**) away from it. Second row: the vertex of the cuboctahedral NP with the long edge of the rectangle (**c**) parallel and (**d**) anti-parallel to the bond of atoms C1 and C2.

We explored two different symmetrical orientations for each NP isomer with a vertex on a vacancy, as shown in Figure 6. In the icosahedral and decahedral NPs, a pentagon is formed by the atoms of the atomic layer behind the vertex tip. In the orientation of Figure 6a the tip of this pentagon points towards the lone C3 atom; in the orientation of Figure 6b, it points away from it. For the cuboctahedral NP the orientations relate to the rectangle formed by the atoms of the second atomic layer of the cuboctahedral NP. The long edge of this rectangle is either parallel to the bond of atoms C1 and C2, orientation of Figure 6c, or anti-parallel to it, orientation of Figure 6d. 

The corresponding binding energy results after relaxation are shown in Table 2. For the decahedral and icosahedral NPs, the orientation of Figure 6b is slightly preferred energetically, possibly because three Au atoms (Au2, Au3, and Au6) sit directly on top of C atoms. However, the energy difference is minimal, only 0.001–0.002 meV. For the cuboctahedral NP, we observe an energy difference of 0.06 eV between the two orientations, with the orientation shown in Figure 6c being the lower energy one. The binding energies of the NPs with a vertex on pristine graphene and on a graphene vacancy are compared in Table 2. For all three structural motifs, the presence of the vacancy strengthens the bonding of the NPs on graphene by several eVs. Moreover, although few Au atoms interact with the C atoms-mainly the 1 atom at the NP tip and, secondly, the 4/5 atoms of the layer after it, the binding energies are comparable to the (111) facet-on-graphene cases.

The addition of the NP at the vacancy site was found to remove the Jahn-Teller distortion in the C atoms surrounding the vacancy. An example of a vertex-on-graphene case is shown in Figure 3b. The three C atoms are pushed away from the cluster tip and the distances between them are on average 2.70 Å. The charge is equally distributed among the three C atoms and the magnetic moments disappear. In general, the in-plane displacement of the C atoms in the neighborhood of the vacancy is on the 0.1 Å scale. In the vertex-on-graphene case, the C atoms around the vacancy are displaced out of plane by 0.1–0.3 Å. The NPs easily absorb the strain induced on the Au surface in the facet-on-graphene case and, except for the first and second layers, are almost unaffected. This minimal strain of the NPs is in agreement with previous studies [16]. In the facet-on-graphene case, the equilibrium distance between the NPs and the graphene sheet is 3.30 Å, the same value previously reported in the literature [11]. In the vertex on graphene vacancy case, the corresponding distance is 1.60 Å, close to the value calculated in the literature for atomic adsorption of Au on a single graphene vacancy [34].

**Figure 7:**
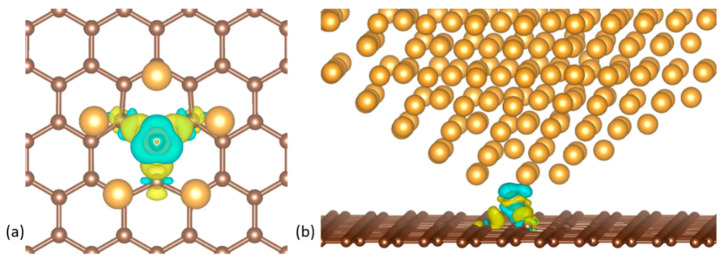
(**a**) Top and (**b**) side views of the charge density difference of a decahedral Au_561_ nanocluster with a vertex on a graphene vacancy. Blue and yellow colors represent charge depletion and accumulation, respectively.

The charge density difference Δρ, shown in Figure 7 for a decahedral NP on a vacancy, was calculated according to:

(3) $$\Delta \rho =\rho_{cluster+graphene}-\rho_{cluster}-\rho_{graphene},$$

Where $\rho_{cluster+graphene}$, $\rho_{cluster}$ and $\rho_{graphene}$, are the electron charge densities obtained from static runs of the relaxed models of the NP on graphene, the graphene sheet and the free NP, respectively. We observed a small charge transfer to the two C atoms bonded in the Jahn-Teller distortion of 0.14 e and from the third lone C atom of 0.04 e. There is also a charge transfer to the Au atom at the tip of the NP of 0.23–0.24 e. Visible in Figure 7a, is the uniform charge distribution between the three C atoms interacting with the NP tip. The charge density difference in Figure 7b illustrates the striking fact that only one Au atom essentially participates in the bond between the NP and the graphene sheet. This also explains the lack of any strain or deformations on the whole NP in the vertex-on-graphene cases.

**Figure 8:**
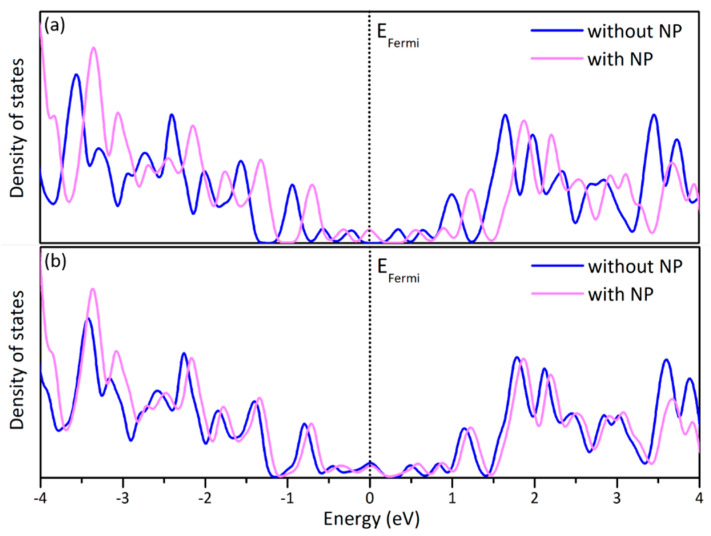
The partial density of states of the C atoms of (**a**) a pristine graphene sheet with and without an icosahedral Au NP oriented with a (111) facet parallel to the graphene sheet and (**b**) a graphene sheet with a vacancy with and without an icosahedral Au NP oriented with its vertex on the vacancy.

The electronic properties of graphene are affected by the presence of the NPs. In Figure 8, the partial density of states (DOS) of graphene sheets sampled at the Γ-point with and without Au NPs is shown. The greater effect is observed in the facet-down case. In Figure 8a a comparison between pristine graphene and pristine graphene interfaced with the (111) facet of an icosahedral Au NP is shown. All peaks are “shifted” towards higher energies by ~0.2 eV when the NP is added. When an icosahedral NP is placed with a vertex on a graphene vacancy, the levels before and after the peak of the Fermi level are again “shifted” towards higher energies by ~0.1 eV (Figure 8b).

Finally, the energetic ordering of the NPs is unchanged by the interaction with the graphene sheet. The stability of the bare NPs follows the icosahedral > decahedral > cuboctahedral trend reported previously in previous theoretical studies of regular clusters in the literature [35], with an energy difference of 2.97 eV to the decahedral and 6.83 eV to the cuboctahedral NP from the icosahedral isomer, respectively. These energy differences become 3.68 eV and 4.25 eV, respectively, when the NPs are accommodated with their (111) facet on graphene (3.51 eV and 4.49 eV with a C vacancy under the NP facet); and to 3.26 eV and 7.14 eV when the NPs are pinned with their vertices in a graphene vacancy. We note that the experimental measurements of the isomers’ relative stability differ from the theory predictions for regular clusters [36].

## 4. Discussion

We investigated the energetics and the electronic, magnetic, and structural effects of graphene vacancies on Au NP-graphene hybrid nanostructures in this work. To do so we used large-scale models consisting of more than 10^3^ atoms in ab initio calculations that took into account spin-polarisation and van der Waals interactions. Our results showed that defects enhance the bonding of Au_561_ clusters with three atomic structures on graphene, particularly when the clusters sit with a vertex on the vacancy. The resulting binding energies in the last case were similar to the facet-on-graphene case, despite the involvement of only 1 Au atom in the bond with graphene, compared with 21 in the parallel-facet case. All of the resulting nanostructures showed a small strain, a symmetrical displacement of the C atoms in the immediate neighborhood of the vacancy, a uniform distribution of the charge between these C atoms, and a minimal strain on the NPs. The increased binding energy and the competition between facet-down and vertex-down are expected to be even more pronounced in clusters smaller than 561 atoms, since fewer atoms belong to the (111) facets. In contrast, the number of atoms in the vertex is independent of the NP size. We generally expect the vertex-on-graphene binding energies to remain constant with the size of the NPs, and the facet-on-graphene binding energies to increase with the size of the NPs.

We believe that a closer look into the electronic properties of these nanostructures would be of great interest for future studies and critical for their application in electronic devices. The methodology of this paper can also be applied for the examination of the properties of another novel hybrid nanostructure of NPs and graphene: graphene-covered gold NPs [37,38]. Such a study should consider the structural changes that might appear in both graphene and the nanoparticles [39]. Experimental information, such as high-resolution electron microscopy images, would be of the utmost importance for replicating the realized nanoparticles in such a theoretical study.

## Figures and Tables

**Table 1:** The binding energies (eV) of the Au_561_ NPs oriented with their (111) surface parallel to the graphene plane with and without a vacancy.

NP Shape	Pristine Graphene	Graphene with Vacancy
Cuboctahedral	−5.81	−5.98
Decahedral	−6.52	−6.74
Icosahedral	−6.90	−7.14

**Table 2:** The binding energies (eV) of the Au_561_ NPs are oriented with a vertex on pristine graphene and on a graphene vacancy.

NP Shape	Pristine Graphene	Graphene with Vacancy
Cuboctahedral	−2.14	−5.21
Decahedral	−2.53	−5.51
Icosahedral	−2.51	−5.52

## References

[1] Li Q., Mahmood N., Zhu J., Hou. Y., Sun. S. (2014). Graphene and its Composites with Nanoparticles for Electrochemical Energy Applications. Nano Today.

[2] Yin P.T., Shah S., Chhowalla M., Lee K.B. (2015). Design, Synthesis, and Characterization of Graphene-nanoparticle Hybrid Materials for Bioapplications. Chem. Rev..

[3] Yeh Y.C., Crerana B., Rotelloa V.M. (2012). Gold Nanoparticles: Preparation, Properties, and Applications in Bionanotechnology. Nanoscale.

[4] Elahi N., Kamali M., Baghersad M.H. (2018). Recent Biomedical Applications of Gold Nanoparticles: A Review. Talanta.

[5] Thompson D.T. (2007). Using Gold Nanoparticles for Catalysis. Nano Today.

[6] Hutchings G.J., Edwards J.K. (2012). Chapter 6—Application of gold nanoparticles in catalysis. Frontiers of Nanoscience.

[7] Muszynski R., Seger B., Kamat P.V. (2008). Decorating Graphene Sheets with Gold Nanoparticles. J. Phys. Chem. C.

[8] Xue Y., Zhao H., Wu Z., Li X., He Y., Yuan Z. (2011). The Comparison of Different Gold Nanoparticles Graphene Nanosheets Hybrid Nanocomposites in Electrochemical Performance and the Construction of a Sensitive Uric Acid Electrochemical Sensor with Novel Hybrid Nanocomposites. Biosens. Bioelectron.

[9] Huang J., Tian J., Zhao Y., Zhao S. (2015). Ag-Au Nanoparticles Coated Graphene Electrochemical Sensor for Ultrasensitive Analysis of Carcinoembryonic Antigen in Clinical Immunoassay. Sens. Actuators B-Chem..

[10] Torres-Mendieta R., Ventura-Espinosa D., Sabater S., Lancis J., Mínguez-Vega G., Mata J.A. (2016). In Situ Decoration of Graphene Sheets with Gold Nanoparticles Synthetized by Pulsed Laser Ablation in Liquids. Sci. Rep..

[11] Giovannetti G., Khomyakov P.A., Brocks G.V., Karpan M., van den Brink J., Kelly P.J. (2008). Doping Graphene with Metal Contacts. Phys. Rev. Lett..

[12] Sundaram R.S., Steiner M., Chiu H.Y., Engel M., Bol A.A., Krupke R., Burghard M., Kern K., Avouris P. (2011). The Graphene-gold Interface and its Implications for Nanoelectronics. Nano Lett..

[13] La Torre A., Gimenez-Lopez M.d.C., Fay M.W., Herreros Lucas C., Brown P.D., Khlobystov A.N. (2015). Dynamics of Gold Nanoparticles on Carbon Nanostructures Driven by Van Der Waals and Electrostatic Interactions. Small.

[14] Claeyssens F., Pratontep S., Xirouchaki C., Palmer R.E. (2006). Immobilization of Large Size-selected Silver Clusters on Graphite. Nanotechnology.

[15] Rodríguez-Zamora P., Yin F., Palmer R.E. (2014). Enhanced Immobilization of Gold Nanoclusters on Graphite. J. Phys. Chem. A.

[16] De la Rosa-Abad J.A., Soldano G.J., Mejía-Rosales S.J., Mariscal M.M. (2016). Immobilization of Au Nanoparticles on Graphite Tunnels Through Nanocapillarity. RSC Adv..

[17] Pulido A., Boronat M., Corma A. (2011). Theoretical Investigation of Gold Clusters Supported on Graphene Sheets. New J. Chem..

[18] Kresse G., Furthmüller J. (1996). Efficiency of Ab-initio Total Energy Calculations for Metals and Semiconductors Using a Plane-wave Basis Set. Comput. Mater. Sci..

[19] Kresse G., Furthmüller J. (1996). Efficient Iterative Schemes for Ab initio Total-energy Calculations Using a Plane-wave Basis Set. Phys. Rev. B.

[20] Perdew J.P., Burke K., Ernzerhof M. (1996). Generalized Gradient Approximation Made Simple. Phys. Rev. Lett..

[21] Blöchl P.E. (1994). Projector Augmented-wave Method. Phys. Rev. B.

[22] Kresse G., Joubert D. (1999). From Ultrasoft Pseudopotentials to the Projector Augmented-wave Method. Phys. Rev. B.

[23] Wells D.M., Rossi G., Ferrando R., Palmer R.E. (2015). Metastability of the Atomic Structures of Size-selected Gold Nanoparticles. Nanoscale.

[24] Grimme S., Antony J., Ehrlich S., Krieg H. (2010). A Consistent and Accurate Ab initio Parametrization of Density Functional Dispersion Correction (DFT-D) for the 94 Elements H-Pu. J. Chem. Phys..

[25] Grimme S., Ehrlich S., Goerigk L. (2011). Effect of the Damping Function in Dispersion Corrected Density Functional Theory. J. Comput. Chem..

[26] Rêgo C.R.C., Oliveira L.N., Tereshchuk P., Da Silva J.L.F. (2015). Comparative Study of Van Der Waals Corrections to the Bulk Properties of Graphite. J. Phys. Cond. Matter.

[27] Buimaga-Iarinca L., Morari C. (2014). Adsorption of Small Aromatic Molecules on Gold: A DFT Localized Basis Set Study Including Van Der Waals Effects. Theor. Chem. Acc..

[28] Rodriguez-Reyes J.C.F., Siler C.G.F., Liu W., Tkatchenko A., Friend C.M., Madix R.J. (2014). Van Der Waals Interactions Determine Selectivity in Catalysis by Metallic Gold. J. Am. Chem. Soc..

[29] Fernández E.M., Balbás L.C. (2011). GGA Versus Van Der Waals Density Functional Results for Mixed Gold/mercury Molecules and Pure Au and Hg Cluster Properties. Phys. Chem. Chem. Phys..

[30] Reimers J.R., Ford M.J., Marcuccio S.M., Ulstrup J., Hush N.S. (2017). Competition of Van Der Waals and Chemical Forces on Gold–sulfur Surfaces and Nanoparticles. Nat. Rev. Chem..

[31] Momma K., Izumi F. (2011). VESTA 3 for Three-dimensional Visualization of Crystal, Volumetric and Morphology Data. J. Appl. Crystallogr..

[32] Zenodo.

[33] Skowron S.T., Lebedeva I.V., Popov A.M., Bichoutskaia E. (2015). Energetics of Atomic Scale Structure Changes in Graphene. Chem. Soc. Rev..

[34] Pašti I.A., Jovanović A., Dobrota A.S., Mentus S.V., Johansson B., Skorodumova N.V. (2018). Atomic Adsorption on Graphene with a Single Vacancy: Systematic DFT Study Through the Periodic Table of Elements. Phys. Chem. Chem. Phys..

[35] Li H., Li L., Pedersen A., Gao Y., Khetrapal N., Jónsson J., Zeng X.C. (2015). Magic-number Gold Nanoclusters with Diameters From 1 to 3.5 nm: Relative Stability and Catalytic Activity for CO Oxidation. Nano Lett..

[36] Foster D.M., Ferrando R., Palmer R.E. (2018). Experimental Determination of the Energy Difference Between Competing Isomers of Deposited Size-selected Gold Nanoclusters. Nat. Commun..

[37] Piszter G., Kertész K., Molnár G., Pálinkás A., Deák A., Osváth Z. (2019). Vapour Sensing Properties of Graphene-covered Gold Nanoparticles. Nanoscale Adv..

[38] Farokhnezhad M., Esmaeilzadeh M. (2019). Graphene Coated Gold Nanoparticles: An Emerging Class of Nanoagents for Photothermal Therapy Applications. Phys. Chem. Chem. Phys..

[39] Osváth Z., Deák A., Kertész K., Molnár G., Vértesy G., Zámbó D., Hwang C., Biróac L.P. (2015). The Structure and Properties of Graphene on Gold Nanoparticle. Nanoscale.

